# Bilateral primary synovial chondromatosis in the knee joint

**DOI:** 10.1002/ccr3.6618

**Published:** 2022-12-12

**Authors:** Mahan Shafie, Zahra Babaei Aghdam, Zeinab Shirzad Moghaddam, Mohammad Ayati Firoozabadi, Seyedeh Zahra Emami Razavi, Maryam Hosseini, Mohaddeseh Azadvari

**Affiliations:** ^1^ School of Medicine Tehran University of Medical Sciences Tehran Iran; ^2^ Department of Radiology, Imaging Sciences Research Group Tabriz University of Medical Sciences Tehran Iran; ^3^ Department of Orthopedic Surgery, Joint Reconstruction Research Center Tehran University of Medical Sciences Tehran Iran; ^4^ Physical Medicine and Rehabilitation Department, Imam Khomeini Hospital Complex Tehran University of Medical Sciences Tehran Iran; ^5^ Urology Research Center Tehran University of Medical Sciences Tehran Iran

**Keywords:** knee locking, knee pain, loose body, synovial chondromatosis

## Abstract

Primary synovial chondromatosis is a disorder characterized by the metaplasia of the synovial membrane and the formation of loose bodies floating in the joint. We described a 30‐year‐old woman without any past medical history complaining of bilateral progressive knee pain who was later discovered to have bilateral synovial chondromatosis.

## Graphical abstract

Synovial chondromatosis is a rare condition and it could be easily misdiagnosed which may lead to the progression of symptoms. Bilateral involvement is also extremely rare; however, further investigations on other joints should be considered.

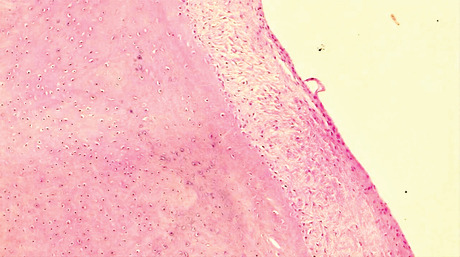

## 1 INTRODUCTION

Primary synovial chondromatosis (SC) is a rare benign proliferative disorder in which the metaplasia of the synovial membrane leads to the formation of clusters of chondrocytes floating in the joint space, which are called loose bodies.^1^ The condition is typically monoarticular and usually affects large joints; however, bilateral or multiple involvements of joints are extremely rare, and usually reported in patients with other underlying articular disorders such as rheumatoid arthritis or osteoarthritis.^2^, ^3^ Patients typically present with joint pain and stiffness; however, the progression of the disease can result in decreased range of motion, effusions, crepitation, and eventual locking of the joint.^3^ Due to non‐specific signs and symptoms especially at the early stages and also the rarity of the disease, SC of the knee is misdiagnosed as meniscal pathology or even osteoarthritis.^1^ Pigmented villonodular synovitis, synovial hemangioma, and lipoma are other conditions that can mimic synovial chondromatosis. It affects men more than women and it is most common in people between the ages of 20 and 40.^4^ However, there are some reports that show that it can be also found in children.^5^ It is commonly intra‐articular but can also lead to extra‐articular proliferation and debilitating disease with cancerous changes in the long run. The disease has three different stages. Synovial intima undergoes metaplasia during phase I in which no calcifications are visible on the synovium, despite active synovitis and nodule formation. Synovitis and cartilaginous loose bodies occur in phase II. However, in phase III, the synovitis disappears but the loose bodies persist, and as well as unifying and calcifying, they tend to condense.^6^ Herein, we described a 30‐year‐old woman without any other past medical history complaining of bilateral long‐lasting progressive knee pain who was later discovered to have bilateral primary SC of both knees.

## 2 CASE PRESENTATION

A 30‐year‐old woman presented with a history of left knee pain in the past three years which got exacerbated by walking or taking stairs. Her pain gradually intensified over years, accompanied by joint locking. The pain was mainly in the popliteal fossa, depriving the patient of extending the knee joint normally. The patient would alleviate the pain and locking by massaging the knee. The symptoms mainly existed in the left knee, but over time, the right knee began to show some slight symptoms as well. At first, she had no limitation in range of motion and the episodes of pain were occasionally and pain usually resolved after resting for a few days. Eventually, when she hopped over a step, there came a sudden locking that would not alleviate anymore by massaging or resting, resulting in referring to the hospital.

She had no history of trauma, other medical conditions, or drug history. There were no similar conditions among her family members, and she had not had any previous procedures done before. Constitutional symptoms including weight loss, fever, fatigue, or malaise were also negative. At the time of admission to the hospital, the patient was independent in terms of daily activities with the ability to walk without assistive devices. On palpation, the popliteal area was tender with mild swelling of the joint filling the suprapatellar pouch. No skin abnormality was observed over the knees. There were five degrees of flexion contracture in the left knee while the right knee range of motion was normal. Meniscus tests were done with normal results in passive flexion, but painful and with muscle guarding on extension. There was no ligamentous laxity and patella was stable and examination of the other joints was unremarkable.

**FIGURE 1:**
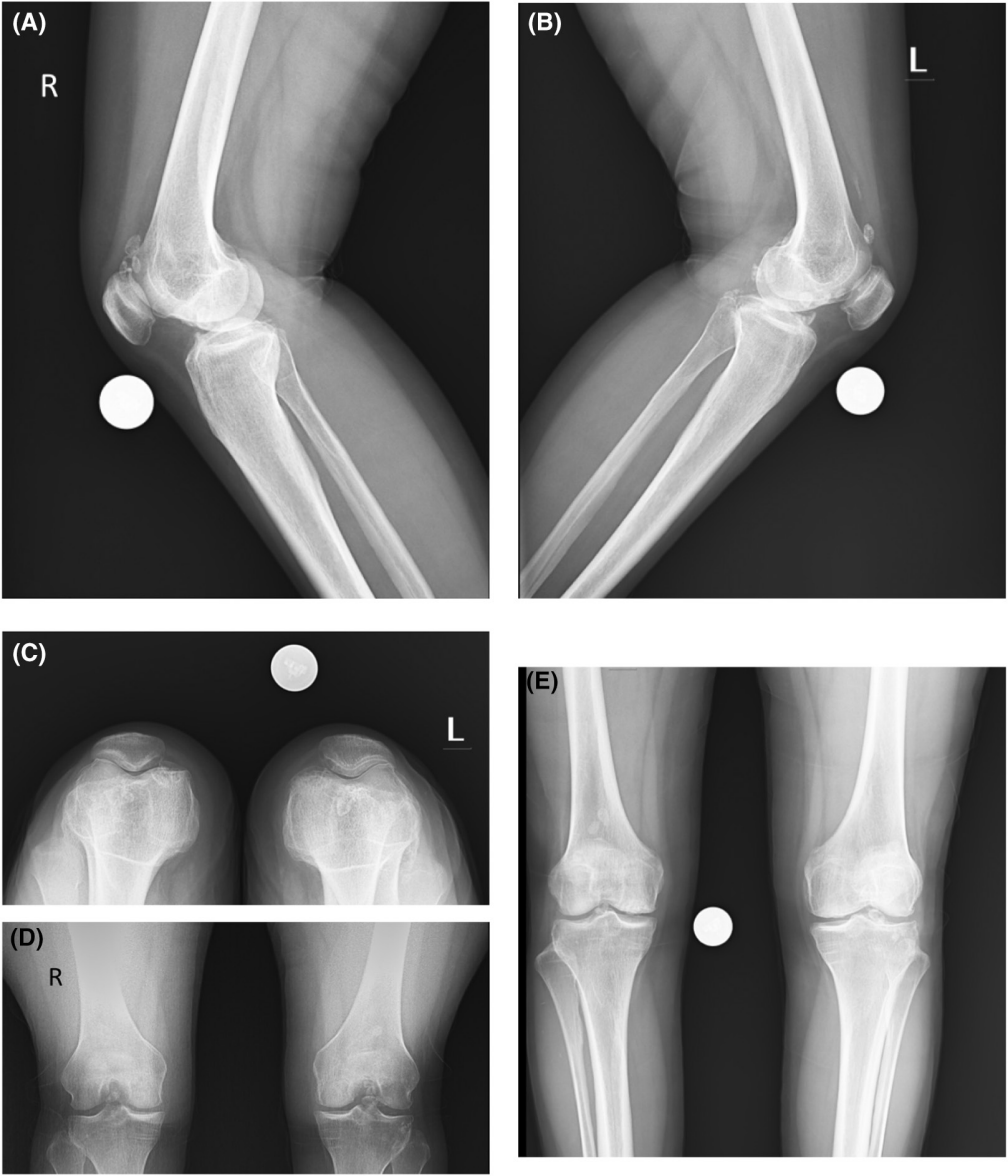
(A) Lateral view of right knee, (B) Lateral view of left knee, (C) Skyline view, (D) Rosenberg view, (E) AP view

**FIGURE 2:**
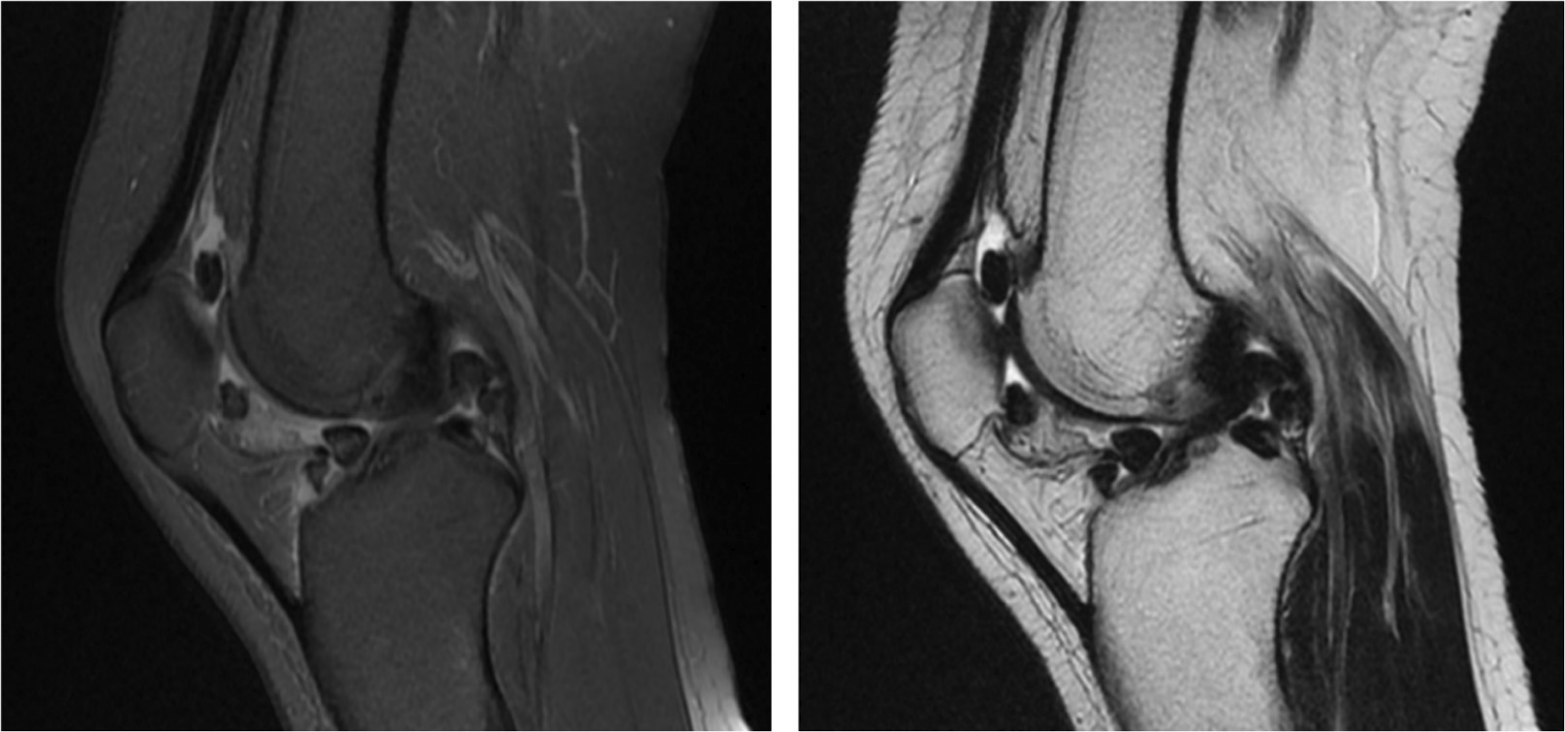
Left Knee MRI

**FIGURE 3:**
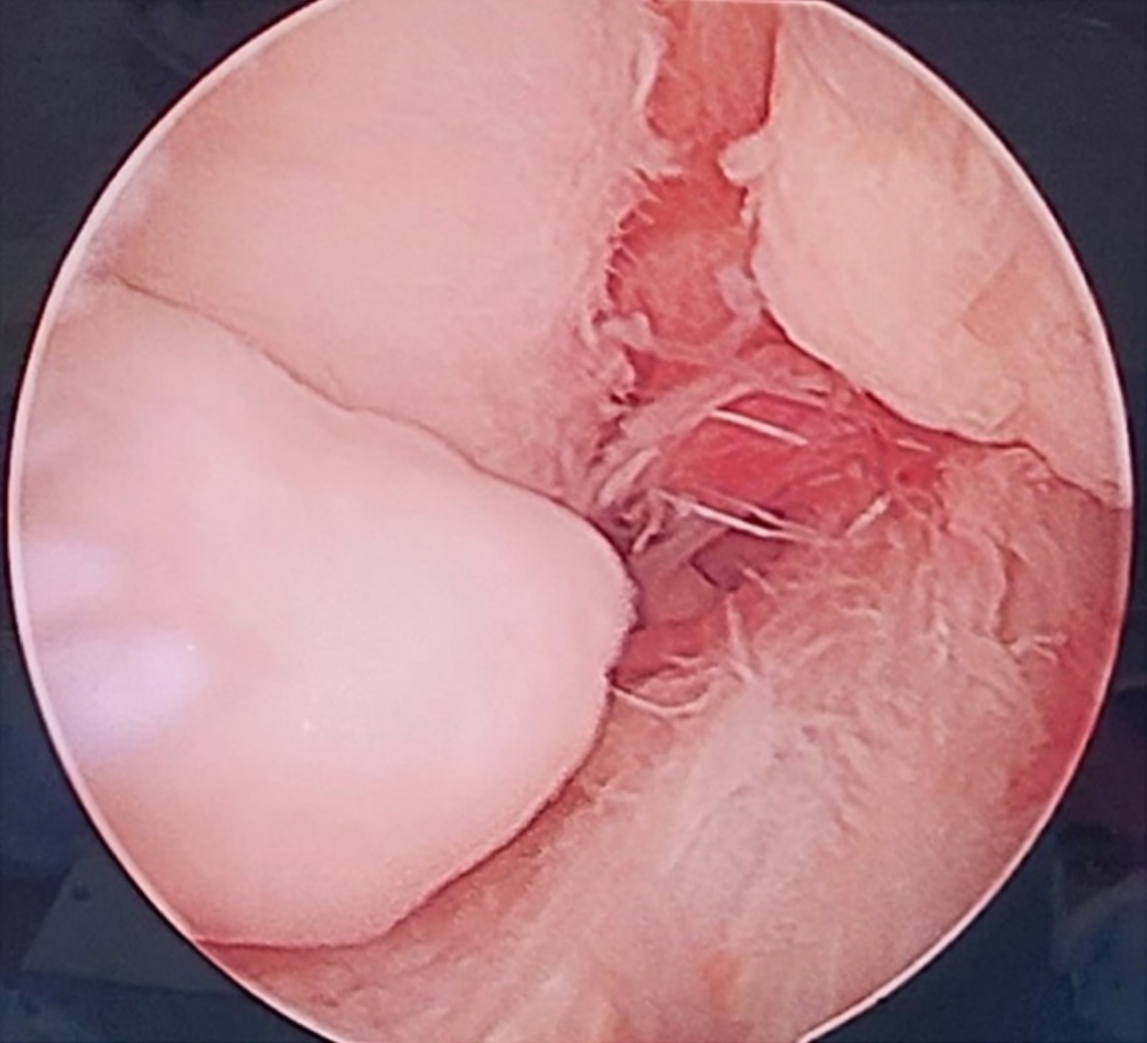
Arthroscopy of the left knee

The patient's blood tests were unremarkable. A plain X‐ray of both knees revealed unspecific multiple calcifications (Figure 1); however, MRI showed multiple loose bodies in the knee joints. MRI reported multiple intra articular intermediate signal lesion in supra patellar, posterior aspect Hoffa's fat pad, and posterior joint capsule up to 8 mm suggestive for synovial chondromatosis, and also focal cartilage loss in medial femoral condyle with subchondral irregularity and cystic changes suggestive of focal degenerative joint disease (Figure 2). Under 250 mm Hg tourniquet pressure, left knee arthroscopy was performed through anteromedial and anterolateral portals. During knee arthroscopy, a white mass with the appearance of cartilage was observed in the anterior compartment of the knee. This mass prevented the full extension of the knee due to contact with the notch and femoral medial condyle. With the help of an arthroscopic shaver, the bed of this lesion was released and with the help of an arthroscopic grasper, it was removed through the anteromedial portal. After removing the mentioned mass, a chondral lesion on the notch and medial femoral condyle caused by the mass contact during the range of motion was evident. Due to the small extent of the lesion (less than one square centimeter), only chondroplasty was performed (Figure 3).

**FIGURE 4:**
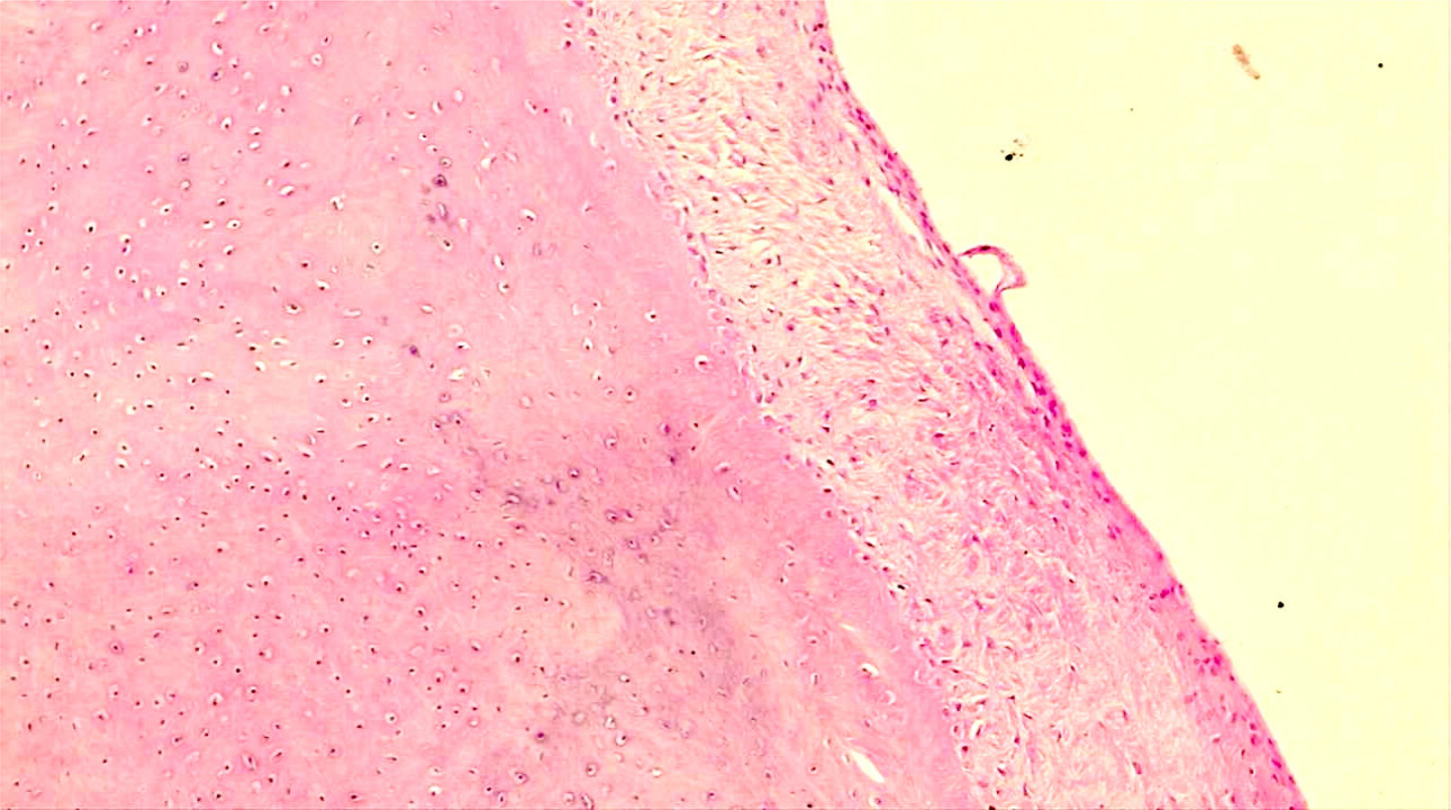
Left knee histopathology

After three months, the right knee was also operated arthroscopically for the removal of loose bodies and synovectomy. The pathology reported cartilaginous lesion with no obvious nuclear atypia and without atypical changes. The sections showed clusters of chondrocytes with focal ossification and focally attenuated synovium overlying the nodules. Thus, histologic findings could be compatible with intraarticular loos body in an appropriate clinical and radiographic setting, all in favor of synovial chondromatosis (Figure 4).

After each arthroscopy, an early active assistive range of motion (ROM) was initiated for the patient, and in a six‐month follow‐up, she gained a full normal range of motion for each knee. Symptoms resolved significantly, and the patient was able to walk without any assistance. The force of the quadriceps muscle was 4 out of 5 on both sides.

## 3 DISCUSSION

Primary SC, also called osteochondromatosis, is a rare benign metaplastic disease of the synovial tissue and in the majority of cases is a monoarticular disorder affecting large joints. In this case report, the patient was a female in her 30s suffering from knee pain for about three years, with palpable nodules around the left knee and interlocking symptoms that all got severe just before she sought medical attention. She also reported pain in the opposite knee at evaluation, which was later discovered to share the same pathology. It is noteworthy, the bilateral involvement of joints is extremely rare, which is usually reported in patients with other articular disorders such as rheumatoid arthritis or osteoarthritis; however, our patient reported no other underlying disease.^2^, ^3^ She also reported no previous trauma, and meniscal injury or ligamentous tears were ruled out. Due to her occupation, she was often forced to stand for extended periods of time; therefore, early onset osteoarthritis was one of the main differential diagnoses. Other types of arthritis were also on the list but the presence of loose bodies on the MRI and the report of pathology confirmed the diagnosis of SC in both knees. This patient has tried to reduce the pain and interlocking symptoms occasionally by moving and manipulating the nodules. This has led to more articular damage making the operation and post‐op follow‐ups even more important. The palpated masses are often the result of a long and neglected disease process^7^; likewise in our case, the first symptoms arose about three years ago.

A pathognomic radiologic sign of SC is the presence of loose bodies which is also the most common reported radiologic sign in the literature, however, there are reported cases of pathologically confirmed SC without evident radiologic loose bodies. This could be due to the small number of intra‐articular radiolucent bodies or even the absence of loose bodies in the early stages of the disease when only active lesions of the synovium are present.^8^, ^9^ In such circumstances when SC is suspected, MRI should be considered. Synovial proliferation, erosive reactions, and joint effusion are commonly seen on MRI. Loose bodies present as low to intermediate signal and high signal bodies on T1‐ and T2‐weighted MRI, respectively. Occasionally, they can appear low signal as a result of extensive calcification. The final confirmatory diagnostic tool is histologic evaluation, compatible with a benign cartilaginous proliferation.^10^, ^11^

In addition to its rarity, biarticular involvement is even more uncommon and to the best of our knowledge, there have been only four cases of bilateral knee involvement in the literature. One of them was found in an 18‐year‐old boy with accompanying genu valgum deformity.^3^ Tahmasebi et al.^2^ reported the same involvement in a 34‐year‐old female patient with underlying rheumatoid arthritis. The other two cases were reported in 38‐ and 68‐year‐olds with no other prior pathology.^12^, ^13^ Same as our case, in all other four patients, palpable masses and restricted range of motion were identified on clinical examination. The involvement was both intra‐ and extra‐articular in all cases and calcified densities were present in radiographic investigations of all patients. It could be suggested that bilateral involvement happens in severe cases and is an indicator of high‐stage disease requiring more accurate and invasive treatment. Also, it is important to keep in mind that biarticular involvement cannot rule out SC.

In the first stages of the disease, before the presence of loose bodies, conservative treatments such as NSAID use, intraarticular injections, and bracing are more warranted. However, as the metaplastic changes progress and free bodies appear causing limited range of motion and diffuse swelling, surgical interventions, arthrotomy, and synovectomy become inevitable. Although open surgical interventions are done if there is suspicion of malignancy or severe articular damage which needs a total knee replacement, arthroscopy has been proposed as the choice of surgical method in most cases.^14^ Some surgeons decide to only remove the loose bodies, and some prefer to also do a synovectomy. However, if only the loose bodies are removed, recurrence is possible. If relapse occurs, synovectomy should be done and for refractory severe cases, radiation has also been suggested.^15^ Radio synovectomy could be considered as an adjuvant before the operation to minimize the damage to articular surfaces during surgery and to further prevent osteoarthritis, which is a known adverse effect of multiple operations in SC.^4^, ^16^ Nevertheless, the increased risk of malignancy should be calculated when choosing radiation treatment. Even after successful treatment, close follow‐ups are needed not only for refractory disease but also to look out for malignant changes.^17^

In this case, it is not clear whether our subject would have benefited from more extensive and invasive management such as arthrotomy which is usually reserved for advanced stages.^2^ Also, the long‐term outcome of different diagnostic approaches and interventions is not comparable in a such case report. Hence, we recommend reviewing the reported cases considering various diagnostic planning, treatment, and short‐ and long‐term outcomes, and also investigating these issues in comparative and interventional studies to reach a consensus in the clinical diagnosis and surgical management of SC.

Knee pain with swelling may be caused by various conditions ranging from a simple muscle strain to articular and soft tissue malignant disease; however, in an adult in the absence of past trauma without proper response to conservative treatments, the possibility of SC should be kept in mind. As SC is a rare condition, it could be easily misdiagnosed which may lead to the progression of symptoms, and its progressive dissemination into the articular structures will result in joint destruction. Bilateral or multiple involvements are also extremely rare; however, further investigations on other joints should be considered. It should be noted that conventional X‐ray cannot exclude SC and neither a bilateral involvement nor MRI is the choice of diagnostic modality. Early diagnosis with early interventions can prevent the disease from progressing to such debilitating disease in our case.

## AUTHOR CONTRIBUTIONS

MS contributed to developing the research idea, composing, and revising the manuscript. ZB contributed to composing and revising the manuscript. ZS contributed to composing and revising the manuscript. MA contributed to developing the research idea and revising the manuscript. SE contributed to developing the research idea and revising the manuscript. MH contributed to developing the research idea and revising the manuscript. MA contributed to developing the research idea and revising the manuscript.

## CONFLICT OF INTEREST

The authors have no conflict of interest to declare.

## FUNDING INFORMATION

This work did not receive any specific grant from any funding agency in the public, commercial, or not‐for‐profit sectors.

## ETHICAL APPROVAL

This study was approved by the research and ethics committee of Tehran University of Medical Sciences. The patient has given her informed consent to publish this case.

## CONSENT

Written informed consent was obtained from the patient for publication of this case report and any accompanying images. A copy of the written consent is available for review by the Editor‐in‐Chief of this journal.

## Data Availability

Data sharing is not applicable to this article as no datasets were generated or analyzed during the current study.
